# Shape Effects of Peptide Amphiphile Micelles for Targeting Monocytes

**DOI:** 10.3390/molecules23112786

**Published:** 2018-10-27

**Authors:** Johan Joo, Christopher Poon, Sang Pil Yoo, Eun Ji Chung

**Affiliations:** 1 Department of Biomedical Engineering, University of Southern California, Los Angeles, CA 90089, USA; johanjoo@usc.edu (J.J.); poonc@usc.edu (C.P.); 2 Institute for Molecular Engineering, University of Chicago, 5747 South Ellis Avenue, Chicago, IL 60637, USA; sangpilyoo@mednet.ucla.edu; 3 Current Affiliation: Medical Scientist Training Program, David Geffen School of Medicine, University of California, Los Angeles, CA 90096, USA; 4 Mork Family Department of Chemical Engineering and Materials Science, University of Southern California, Los Angeles, CA 90089, USA; 5 Division of Nephrology and Hypertension, Department of Medicine, Keck School of Medicine, University of Southern California, Los Angeles, CA 90033, USA; 6 Norris Comprehensive Cancer Center, University of Southern California, Los Angeles, CA 90033, USA; 7 Department of Stem Cell Biology and Regenerative Medicine, University of Southern California, Los Angeles, CA 90033, USA

**Keywords:** monocytes, peptide amphiphile micelles, monocyte targeting, nanoparticle shape

## Abstract

Peptide amphiphile micelles (PAMs) are a nanoparticle platform that have gained popularity for their targeting versatility in a wide range of disease models. An important aspect of micelle design is considering the type of hydrophobic moiety used to synthesize the PAM, which can act as a contributing factor regarding their morphology and targeting capabilities. To delineate and compare the characteristics of spherical and cylindrical micelles, we incorporated the monocyte-targeting chemokine, monocyte chemoattractant protein-1 (MCP-1), into our micelles (MCP-1 PAMs). We report that both shapes of nanoparticles were biocompatible with monocytes and enhanced the secondary structure of the MCP-1 peptide, thereby improving the ability of the micelles to mimic the native MCP-1 protein structure. As a result, both shapes of MCP-1 PAMs effectively targeted monocytes in an in vitro binding assay with murine monocytes. Interestingly, cylindrical PAMs showed a greater ability to attract monocytes compared to spherical PAMs in a chemotaxis assay. However, the surface area, the multivalent display of peptides, and the zeta potential of PAMs may also influence their biomimetic properties. Herein, we introduce variations in the methods of PAM synthesis and discuss the differences in PAM characteristics that can impact the recruitment of monocytes, a process associated with disease and cancer progression.

## 1. Introduction

In the past several decades, the development of peptide-based nanoparticles for applications in medicine have grown due to their targeting versatility, drug delivery potential, and diagnostic capabilities [1,2,3]. By varying the peptide sequence, nanoparticles containing drugs or imaging-agents can target specific cell types in diseases [1,2,4,5,6,7,8]. Peptide amphiphiles (PAs) are a class of molecules in which a peptide headgroup is conjugated to a hydrocarbon tail, resulting in structures that possess distinct hydrophobic and hydrophilic regions [9,10,11]. When PAs are suspended in solution above their critical micelle concentration (CMC), PAs self-assemble into peptide amphiphile micelles (PAMs) partially through hydrophobic interactions that occur at the core of the micelle [4]. One advantage of inducing PA micellization is the enhancement of the secondary structure of the peptides via hydrogen bonding when incorporated within a PAM [1,2,12]. While short, synthetic peptides dispersed in solution lose much of their secondary structure, pairing the peptides with a lipid tail to form micelles can enhance the secondary structure that is characteristic of the endogenous peptide sequence within the full protein [2,12].

The morphology and shape of the self-assembled nanoparticles is in part dependent on the nature of the nonpeptidic component of the PA, which can be described using Israelachvili’s surfactant number theory [13]. This theory describes the critical packing parameter (p) of the monomer to be:

$$p=\frac{v}{a\times l},$$

where *v* represents the volume of the tail, *a* is the area of the headgroup, and *l* is the length of the tail. For instance, DSPE-PEG_2000_ (1,2-distearoyl-*sn*-glycero-3-phosphoethanolamine-*N*-(polyethylene glycol)-2000) have large headgroups compared to their short lipid tails, resulting in p values < 1/3 and the formation of spherical micelles, typically with an average diameter of 10–20 nm [12,14]. On the other hand, the double C_16_ hydrocarbon chain, or diC_16_ (1′,3′-dihexadecyl *N*-succinyl-l-glutamate), possesses a shorter headgroup relative to its lipid tails. Therefore, diC_16_ PAs have been shown to form elongated cylindrical micelles (P values between 1/3 and 1/2) with lengths that can be greater than 1 µm [5,15,16].

Both spherical and cylindrical PAMs have shown promising results in disease applications. Karmali et al. conjugated two tumor-targeting peptides, CREKA and LyP-1 (CGNKRTRGC), with DSPE-PEG_2000_ to target tumors in mice [17]. Subsequent in vivo administration of spherical PAMs resulted in pronounced accumulation at the tumor site. Additionally, Mao et al. synthesized DSPE-PEG_2000_ micelles conjugated with the integrin-binding peptide, iRGD, and loaded the cancer-targeting drug, salinomycin. iRGD micelles exhibited considerable localization and cytotoxicity to liver cancer cells [18]. These studies demonstrate the targeting versatility of spherical micelles that contain DSPE-PEG_2000_ in disease settings.

In addition, cylindrical micelles have also been reported to have advantages in cell targeting and adhesion [15]. Moyer et al. reported that cylindrical PAs containing a collagen-binding peptide sequence (KLWVLPKC) targeted areas of arterial damage [19]. Furthermore, diC_16_ tails conjugated with the tumor suppressing peptide p53_14–29_ exhibited enhanced localization and internalization to SJSA-1 osteosarcoma cells compared to free peptides [16]. In sum, cylindrical micelles, similar to spherical micelles, also demonstrate favorable targeting capabilities.

To further understand and delineate the differences of spherical and cylindrical micelles, we designed DSPE-PEG_2000_ PAMs and diC_16_ PAMs to target monocytes by incorporating the first peptide loop (residues 13–35) of monocyte chemoattractant protein-1 (MCP-1), which was previously found as the binding sequence of the CCR2 receptor and to possess chemotactic abilities [20]. MCP-1 plays a crucial role in the proliferation of monocytes, inflammation, and the pathogenesis of many diseases [21,22,23,24], and spherical MCP-1 PAMs consisting of DSPE-PEG_2000_ were previously reported to target monocytes in a murine model of atherosclerosis and showed potential as nanodiagnostic agents [1,2,4].

Herein, we synthesized and characterized spherical MCP-1 PAMs (S-MCP-1 PAM) and cylindrical MCP-1 PAMs (C-MCP-1 PAMs), quantified the binding activity of the two micelles, and investigated their biocompatibility with murine monocytes. Finally, we compared the chemoattractant capabilities of the micelles to assess their ability to mimic the bioactive function of the native peptide measured through a chemotaxis assay. Our results indicate that the nonpeptidic component of the PA not only determines the overall shape of the micelle, but also can influence the monocyte-recruitment abilities of PAMs.

## 2. Results

### 2.1. Peptide Amphiphile Micelle (PAM) Synthesis and Characterization

**Scheme 1:**
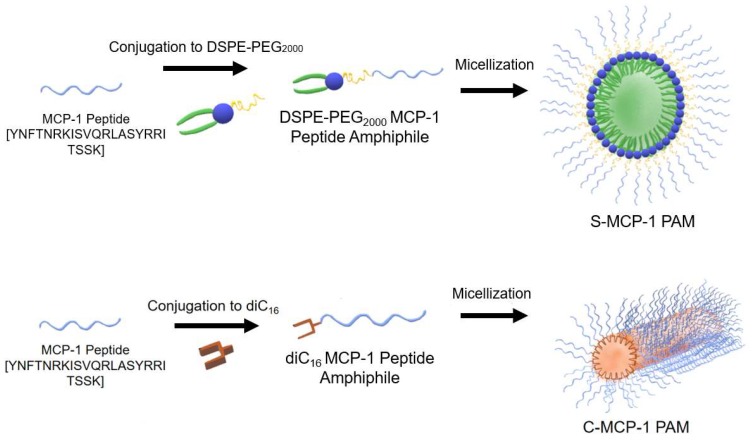
The MCP-1 peptide was conjugated to DSPE-PEG_2000_ to form spherical MCP-1 PAMs, or to diC_16_ tail to form cylindrical MCP-1 PAMs.

The diC_16_ tail was verified with ^1^H-NMR analysis (Figures S2 and S3) before the MCP-1 peptide was conjugated to diC_16_ tail via a peptide bond or DSPE-PEG_2000_-maleimide via a thioether linkage. A scrambled version of the MCP-1 peptide was also synthesized and conjugated to DSPE-PEG_2000_ or diC_16_. Micellization of S-MCP-1 PAMs and C-MCP-1 PAMs was conducted by dry film hydration using water or PBS (Scheme 1).

**Figure 1:**
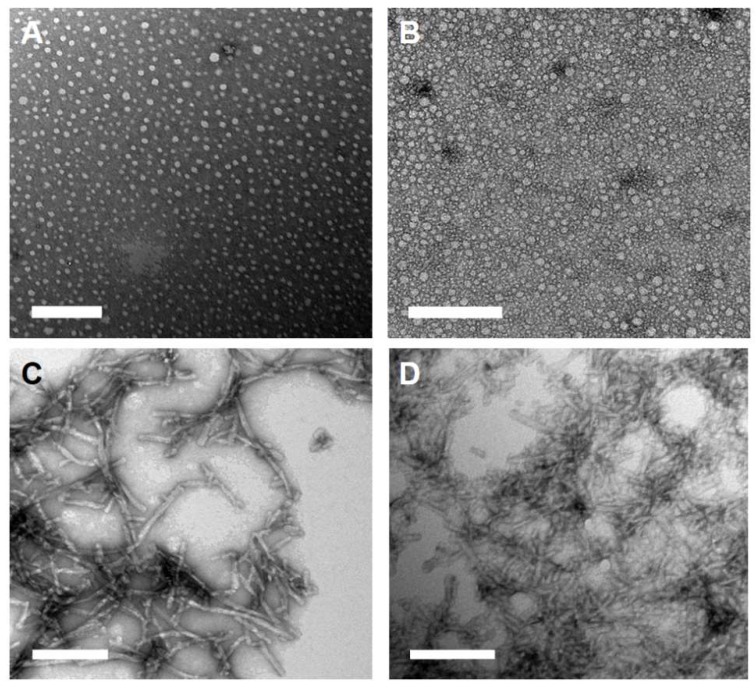
Representative TEM images of (**A**) S-MCP-1 PAMs, (**B**) S-Scrambled PAMs, (**C**) C-MCP-1 PAMs, and (**D**) C-Scrambled PAMs. Scale bar = 200 nm.

**Figure 2:**
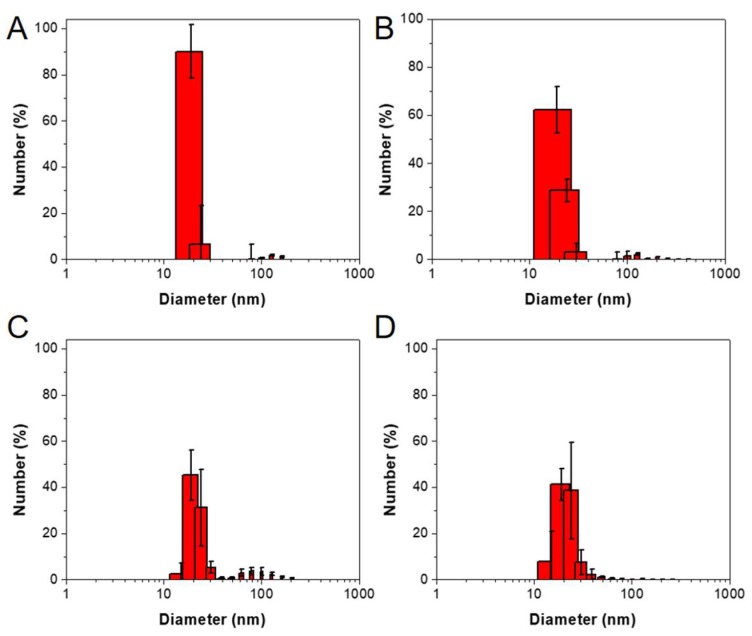
DLS size distributions of (**A**) S-MCP-1 PAMs, (**B**) S-Scrambled PAMs, (**C**) C-MCP-1 PAMs, and (**D**) C-Scrambled PAMs.

Transmission electron microscopy (TEM) confirmed the spherical and cylindrical morphology of micelles with diameters of 14.9 ± 6.0 nm and 15.1 ± 7.1 nm for S-MCP-1 PAMs and S-Scrambled PAMs, respectively (Table 1, Figure 1A–D). Similarly, the diameters of C-MCP-1 PAMs were measured to be 10.7 ± 0.3 nm with lengths of 62.2 ± 41.3 nm, while C-Scrambled PAMs had diameters of 9.3 ± 2.0 nm with lengths of 51.7 ± 15.8 nm (Figure 1C–D, Table 1, Figure 2). In addition, as determined by dynamic light scattering (DLS), the diameters of S-MCP-1 PAMs and S-Scrambled PAMs were 18.7 ± 4.0 nm and 18.4 ± 5.8 nm, respectively, while the diameters of C-MCP-1 PAMs and C-Scrambled PAMs were 24.7 ± 6.2 nm and 23.7 ± 1.6 nm, respectively. (Table 1, Figure 2). As shown in Table 1, the S-MCP-1 and S-Scrambled PAMs had zeta potentials of 1.5 ± 0.6 mV and 3.5 ± 0.5 mV, respectively, which were higher than those of C-MCP-1 and C-Scrambled PAMs (17.6 ± 5.7 mV and 18.8 ± 3.4 mV, respectively). In addition, S-MCP-1 and C-MCP-1 PAMs demonstrated an increase in secondary β-sheet composition (50.9% and 55.4%, respectively) relative to free MCP-1 peptides (36.2%), compared to 46.3% and 55.6% β-sheet composition for the S- and C-Scrambled PAMs, respectively (Table 1, Figure S14). It is crucial to note that the scrambled MCP-1 sequence was designed to mimic the secondary structure of the MCP-1 peptide to ensure that the specific peptide sequence is responsible for chemotactic activity, not its physical structure, which explains the similarities in secondary structure between MCP-1 PAMs and scrambled PAMs. Lastly, the random coil composition decreased to 36.8% and 32.6% for S-MCP-1 and C-MCP-1 PAMs, respectively, from 54.5% (Table 1).

The particle stability of S- and C-MCP-1 PAMs in Figure S15 revealed that the diameters of both PAMs in DMEM supplemented with 10% fetal bovine serum (FBS) steadily increased over the 12 h duration, suggesting that FBS adsorbed onto the nanoparticles and contributed to the increases in size. On the contrary, both PAMs in PBS exhibited no significant changes in particle size.

### 2.2. Biocompatibility and Binding to Monocytes

**Figure 3:**
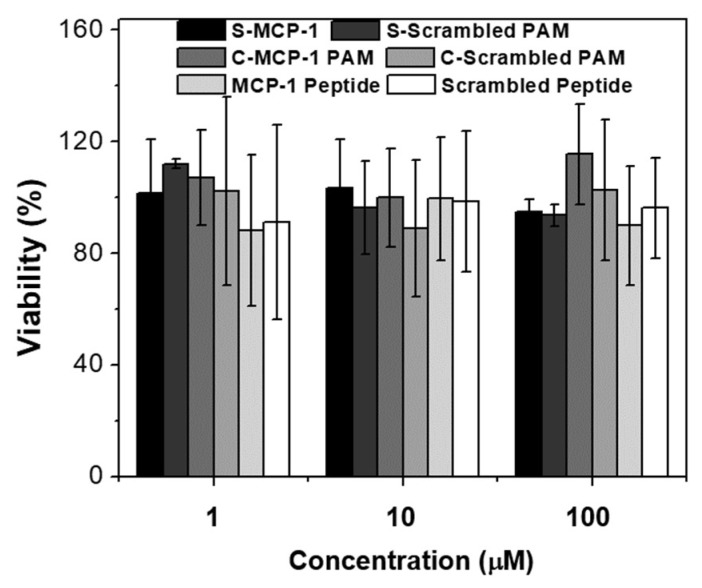
In vitro biocompatibility of S-MCP-1 PAMs, C-MCP-1 PAMs, and scrambled PAMs, free MCP-1 peptides, and scrambled MCP-1 peptides with WEHI-274.1 monocytes after 24 h. Cell viability was normalized to the number of cells treated with PBS in media (100% viability).

**Figure 4:**
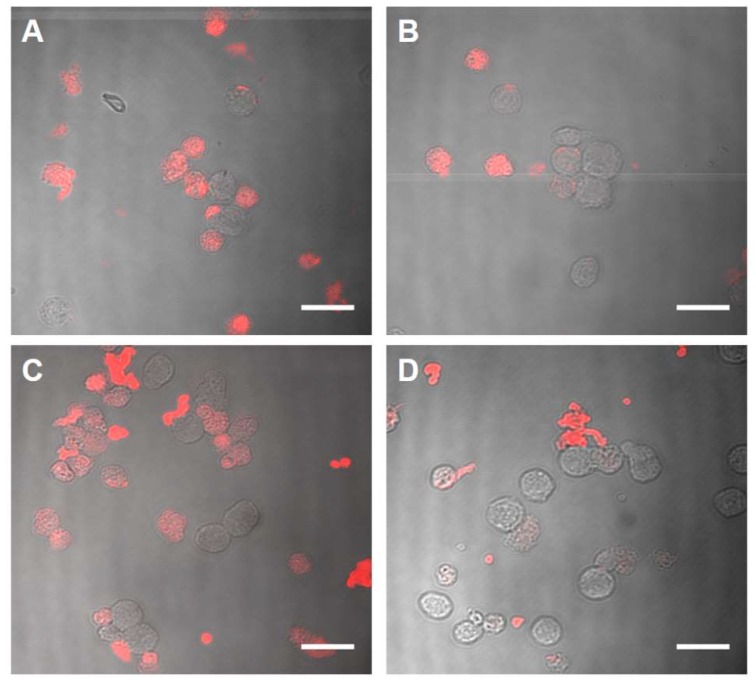
Representative images of (**A**) S-MCP-1-PAMs, (**B**) S-Scrambled PAMs, (**C**) C-MCP-1 PAMs, and (**D**) C-Scrambled PAMs binding to monocytes after 1 h (Scale bar = 20 µm).

In vitro biocompatibility of spherical and cylindrical PAMs was assessed after WEHI-274.1 murine monocytes were incubated with peptides and PAMs for 24 h via MTS assay. No significant decreases in monocyte biocompatibility were found across micelle type, indicating the compatible nature of all PAMs (Figure 3). To quantify binding differences of spherical vs. cylindrical micelles, Cy7-labelled PAMs were incubated with WEHI-274.1 monocytes for 1 h. A significant increase in fluorescence in the S-MCP-1 PAMs compared to the S-Scrambled PAM counterpart was found (Figure 4A,B), which is in agreement with our previous studies [4]. Similarly, the C-MCP-1 PAMs exhibited an increase in binding compared to the C-Scrambled PAM counterpart (Figure 4C,D).

### 2.3. Chemoattractant Properties of PAMs

**Figure 5:**
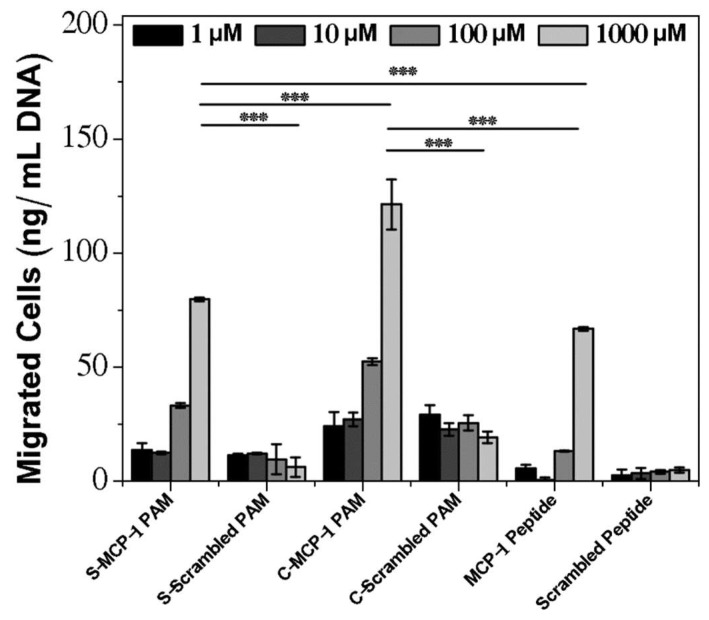
Chemotaxis of murine monocytes after 4 h PAM incubation. Amount of migrated monocyte DNA in the presence of S-MCP-1 PAMs, S-Scrambled PAMs, C-MCP-1 PAMs, C-Scrambled PAMs, and free peptides (n = 4; *** *p* < 0.001).

We assessed the differences in chemoattractant properties of spherical and cylindrical micelles at 1–1000 µM concentrations via chemotaxis assay and measured the total amount of migrated monocyte DNA. As seen in Figure 5, with increasing concentrations of S- and C-MCP-1 PAMs, an increase in chemotaxis activity was found. Specifically, 100 µM and 1000 µM C-MCP-1 PAMs attracted the largest quantities of monocytes (64.7 ± 1.5 ng/mL and 133.6 ± 11.0 ng/mL, respectively). For S-MCP-1 PAMs, the amount of migrated monocytes at 100 µM and 1000 µM was 45.4 ± 1.0 ng/mL and 91.9 ± 0.8 ng/mL, respectively. In contrast, the scrambled versions of all types of micelles showed minimal activity at all concentrations. Overall, C-MCP-1 PAMs exhibited elevated monocyte migration at higher concentrations compared to the S-MCP-1 PAMs. At micelle concentrations below 10 µM, no significant increases in migrated monocytes for both the S and C-MCP-1 PAM groups were found compared to peptides at the same concentrations. Although the free MCP-1 peptide demonstrated a statistically significant monocyte migration with 78.98 ± 0.8 ng/mL at 1000 µM, the amount of DNA present was nevertheless lower than that of both MCP-1 PAM groups. The relative decrease in monocyte DNA for the free MCP-1 peptide suggests that the chemoattractant capabilities of the peptides are improved when encapsulated within a micelle.

## 3. Discussion

Monocytes play a pivotal role in the pathogenesis of numerous diseases such as atherosclerosis and cancer [22,24]. In these disease models, MCP-1 contributes to the recruitment and proliferation of monocytes that leads to the exacerbation of the inflammatory response or the metastasis of cancer cells. Previously, our group demonstrated that MCP-1 PAMs can target monocytes in atherosclerosis [2,4]. However, how the shape of PAMs could be used to influence targeting has not been well elucidated. With this in mind, we engineered S- and C-MCP-1 PAMs to compare and characterize their binding and biomimetic properties.

As residues 13, 28, 30, 34, and 35 are important for the biological activity of the MCP-1 protein, we assessed the secondary structure of the MCP-1 peptide (13–35) to be enhanced within PAMs [21]. As seen in Table 1, our results indicate that the secondary β-sheet structure of the free MCP-1 peptide (residues 13–35) increased significantly when incorporated into S-MCP-1 PAMs and this enhancement in secondary structure is more pronounced when the peptide is placed within C-MCP-1 PAMs. This may be because the tighter packing of cylindrical PAs facilitates the intermolecular hydrogen bonding interactions between peptide head groups [19].

The incorporation of positively charged amino acids (arginine and lysine) in the MCP-1 peptide led to a positive surface charge in both types of PAMs, but to a lesser degree for the S-MCP-1 PAMs [25] (Table 1). This can be attributed to the negatively-charged phosphate group in the DSPE tail that neutralized the positive charges of the peptide [25]. With this in mind, the ability of spherical PAMs to maintain near neutral surface charges may be used to improve bioavailability in vivo and reduce kidney clearance, which are important considerations for in vivo applications [26,27]. It is important to note that the difference in zeta potentials between S- and C- MCP-1 PAMs (Table 1) could also contribute to the binding ability of PAMs to monocytes, as previously reported for monocytes and macrophages [28,29].

As seen in Figure 3, S- and C-MCP-1 PAMs did not impact cell viability, suggesting that both shapes of MCP-1 PAMs do not induce cytotoxic responses, which is consistent with previous reports [2,4]. Interestingly, the binding assay revealed that both S- and C-MCP-1 PAMs demonstrated an increase in monocyte binding compared to the scrambled PAM counterparts (Figure 4). These results indicate that the MCP-1 peptide selectively targets and binds to monocytes. Furthermore, the improved monocyte-binding capabilities of both MCP-1 PAMs could be the result of increased multivalency of MCP-1 peptides on the surfaces of either micelle, thereby facilitating interactions between the peptides and monocytes [19].

Elevated levels of chemoattractant abilities were observed in C-MCP-1 PAMs compared to S-MCP-1 PAMs (Figure 5). These results indicate that C-MCP-1 PAMs show potential to mimic the bioactivity of the MCP-1 protein (*p* < 0.001). The disparity in chemoattractant behavior between the two shapes could be due to the elongated structure and the larger surface area of C-MCP-1 PAMs, which favor the physical interaction between these PAMs and the monocyte receptors as previously mentioned [19]. In addition, the discrepancy in monocyte attraction may also be attributed to the differences in the multivalent display of MCP-1 peptides. The number of DSPE-PEG_2000_ peptide amphiphiles in a single spherical PAM was calculated to be approximately 130 amphiphiles, which is in agreement with other spherical micelles that report between 20 and 300 amphiphiles [30]. In contrast, due to the increase in surface area, cylindrical PAMs can hold up to 4000 peptide amphiphiles [31]. Therefore, the role of peptide multivalency could also enhance the chemoattractant properties of C-MCP-1 PAMs compared to S-MCP-1 PAMs and free peptides. Nonetheless, the free peptides exhibited lower chemoattractant abilities compared to PAMs, which provides further evidence that the ability of either PAMs can enhance the secondary structure of the native protein and mimic the bioactive, functional properties of MCP-1. It is important to note that the chemoattraction is associated with monocyte migration, proliferation, and pro-inflammatory responses that can elicit disease progression in atherosclerosis and prostate cancer [1,2,4,32]. Therefore, it is possible that the enhanced ability of cylindrical PAMs to promote monocyte migration could exacerbate diseased or inflamed areas. However, at the same time, monocytes and monocyte-derived macrophages play a crucial role in disease regression for fibrosis and Alzheimer’s disease [23]. Moreover, by incorporating imaging agents and therapeutics within nanoparticles, monocytes have been used as targets in the diagnosis and treatment of many diseases [2,33,34,35]. Hence, nanoparticle shape may be tailored for specific disease and application contexts.

Given the differences in the surface area, the multivalent display of peptides, and the zeta potentials of spherical and cylindrical MCP-1 PAMs, these properties, in addition to the shape, may contribute to monocyte-targeting and monocyte recruitment. Future work will delineate the contributions of these properties.

## 4. Materials and Methods

### 4.1. Materials

All starting materials were purchased from Sigma Aldrich (St. Louis, MO, USA), unless otherwise noted. DSPE-PEG_2000_-maleimide was purchased from Avanti Polar Lipids (Alabaster, AL, USA). Cy7-amine was purchased from the Lumiprobe corporation (Hunt Valley, MD, USA), and Cy7 mono-*N*-hydroxysuccinimide ester was purchased from GE Healthcare Life Sciences (Pittsburgh, PA, USA). Materials for cell culture, which include fetal bovine serum (FBS, Gibco, Gaithersburg, MD USA), penicillin-streptomycin (Gibco, USA), 2-mercaptoethanol (Gibco, USA), and PBS (Gibco, USA) were purchased from Thermo Fisher Scientific (Waltham, MA, USA). Dulbecco’s Modified Eagle’s Medium (DMEM) was purchased from Sigma Aldrich. WEHI-274.1 murine monocytes (ATCC# CRL-1679) were purchased from ATCC (Manassas, VA, USA) and subjected to mycoplasma testing.

### 4.2. Peptides and Peptide Amphiphile Synthesis

MCP-1 peptides were synthesized according to previous methods outlined by our group [2]. The MCP-1 peptide (residues 13–35; YNFTNRKISVQRLASYRRITSSK) was modified with a cysteine at the N-terminus and synthesized by solid-phase synthesis on Wang resin. A scrambled version of the MCP-1 peptide (YNSLVFRIRNSTQRKYRASIST) was designed to mimic the secondary structure of the MCP-1 peptide. Both peptides were cleaved from the resin using a cleavage solution consisting of a 94:2.5:2.5:1 volume % ratio of trifluoroacetic acid (TFA)–1,2-ethanedithiol–water–triisopropylsilane (TIS). The cleaved peptide was precipitated and washed with diethyl ether and stored at −20 °C DSPE-PEG_2000_-maleimide (11.2 mg) was coupled with the cysteine of MCP-1 peptides (10 mg) through a thioether linkage in 3 mL of water.

1’-3’-dihexadecyl l-glutamate was synthesized through an azeotropic distillation using a Dean-Stark apparatus (Chemglass, Vineland, NJ, USA) by mixing hexadecanol (22.4 g, 0.092 mol), l-glutamic acid (6.8 g, 0.047 mol), and para-toluenesulfonic acid (10.5 g, 0.051 mol) [15] (Figure S1). The crystallized product was purified through Buchner funnel filtration using acetone, and was identified through ^1^H-NMR analysis as shown in Figure S2 (^1^H-NMR in CDCl_3_: 0.88 (t, 6H); 1.26 (m, 55H); 1.52 (m, 4H); 2.18 (tt, 2H), 2.34 (s, 3H), 2.49 (h, 2H), 3.96 (m, 4H), 7.76, 7.12, 7.14, 7.26, 7.73 (dd, 4 H), 8.11, 8.26 (b, 2H). Yield: 83.8%. The diC_16_ tail (1′,3′-dihexadecyl *N*-succinyl-l-glutamate) was synthesized by inserting a spacer molecule, succinic anhydride, in a 1:1 tetrahydrofuran (THF)–chloroform mixture. The resulting product was crystallized in 4 °C overnight and was purified through Buchner funnel filtration with diethyl ether. The purified diC_16_ tail was identified through ^1^H-NMR analysis, as shown in Figure S3 (^1^H-NMR in CDCl_3_: 0.88 (t, 6H), 1.25 (m, 55H), 1.62 (m, 4H), 1.99, 2.17 (tt, 2H), 2.38 (h, 2H), 2.56, 2.69 (tt, 4H), 4.06 (tt, 4H), 4.13, 4.61 (tt, 1H), 6.56 (d, 1H). Yield: 58.4%. The diC_16_ (0.695 g, 1.0 mmol) was coupled to MCP-1 through a peptide bond on resin using *N*,*N*-diisopropylethylamine (218 µL, 1.25 mmol) and *O*-benzotriazole-*N*,*N*,*N*′,*N*′-tetramethyl-uronium-hexafluoro-phosphate (0.4266 g, 1.125 mmol). The coupling reaction was carried out overnight, followed by a cleavage step using a solution of 95:2.5:2.5 volume % ratio of TFA–TIS–water. The product was precipitated and washed with diethyl ether, then stored in −20 ° C. The same procedure was used to synthesize diC_16_ scrambled amphiphiles.

All peptides and PAs were purified through high performance liquid chromatography (HPLC; Shimadzu Corporation, Columbia, MD, USA, Figure S4), and the identity of the purified product was determined through matrix assisted laser desorption/ionization/time-of-flight (MALDI/TOF; Bruker, Billerica, MA, USA) mass spectral analysis. The expected mass peak of both MCP-1 and scrambled MCP-1 is [M + H]^+^ = 2892 (Figures S5 and S6). The expected mass peak of DSPE-PEG_2000_ MCP-1 and DSPE-PEG_2000_ scrambled MCP-1 is [M + H]^+^ = 5830 (Figures S7 and S8). The expected mass peak of diC_16_ MCP-1 and diC_16_ scrambled MCP-1 is [M + H]^+^ = 3571 (Figures S9 and S10).

### 4.3. Fluorescently Labeled Amphiphiles

Fluorescently labeled PAs were synthesized by conjugating Cy7 mono-*N*-hydroxysuccinimide ester to DSPE-PEG_2000_-amine in a 1.1:1 molar ratio. The reaction was carried out in 10 mM aqueous sodium carbonate buffer at a pH of 8.5 for 24 h. The crude product was purified through HPLC on a C4 column, and the collected fractions were identified through MALDI analysis (expected mass peak [M + H]^+^ = 3680) (Figure S11).

Fluorescently labeled diC_16_ amphiphiles were synthesized by conjugating Cy7-amine to the terminal carboxylic acid group of diC_16_ through EDC/NHS chemistry in DMSO with a 1:1.5:4:1:1 molar ratio of diC_16_–Cy7–1-ethyl-3-(3-dimethylaminopropyl)-carbodiimide(EDC)–*N*-hydroxysuccinimide (NHS)–triethanolamine (TEA). The molar amounts of EDC, NHS, and TEA were divided into five aliquots with the first four aliquots added every 2 h while the final aliquot was added 12 h after the previous addition for a total reaction time of 44 h. The crude diC_16_ Cy7 was purified through HPLC purification on a C8 column, and the collected fractions were identified through MALDI analysis (expected molecular weight of diC_16_ Cy7 is [M + H]^+^ = 1326) (Figure S12).

### 4.4. Peptide Amphiphile Micelle (PAM) Assembly

S-MCP-1 PAMs were synthesized by dissolving DSPE-PEG_2000_ MCP-1 or DSPE-PEG_2000_ scrambled MCP-1 and DSPE-PEG_2000_ Cy7 in methanol in a 90:10 molar ratio. This mixture was evaporated using nitrogen, and the resulting dry film was placed under vacuum overnight. The film was hydrated with PBS at 80 °C for 30 min and was cooled to room temperature. C-MCP-1 PAMs using diC_16_ MCP-1 or diC_16_ scrambled MCP-1 and diC_16_ Cy7 were assembled using a similar procedure.

### 4.5. Transmission Electron Microscopy (TEM)

50 µM of PAM solutions in Milli-Q water were placed on carbon grids for two minutes. After wicking away the excess liquid, the grids were washed with Milli-Q water. 1% *w*/*w* phosphotungstic acid was placed on the grids for two minutes, then rinsed with Milli-Q water. To confirm the spherical or cylindrical shapes of the PAMs, dried grids were imaged using JEM 2100-F TEM (JEOL, Ltd., Tokyo, Japan).

### 4.6. Particle Characterization

To quantify the physical properties of spherical micelles, 100 µM of S- and C-MCP-1 PAMs were analyzed using DLS. Measurements were collected at 90° and 637 nm using Dynapro Nanostar system (Wyatt, Santa Barbara, CA, USA). To determine the surface charge of the PAMs, the zeta potentials was determined using a Zetasizer Nano ZS (Malvern, Worcestershire, UK) at 100 µM PAM concentration. The diameters and/or lengths of at least 50 S-MCP-1 PAMs, S-Scrambled PAMs, C-MCP-1 PAMs, or C-Scrambled PAMs were quantified via ImageJ from TEM images.

### 4.7. Particle Stability

S-MCP-1 PAMs and C-MCP-1 PAMs were rehydrated in PBS or DMEM (supplemented with 10% FBS) to make 100 µM PAM solutions. The particle size and distribution were measured every 2 h for a total of 12 h at 37 °C to determine particle stability.

### 4.8. Circular Dichroism

Circular dichroism (CD) spectroscopy was carried out using a Jasco J-815 spectropolarimeter (Easton, MD, USA). PAM solutions (100 µM) were analyzed in 0.2 mm path-length cuvettes. Measurements were made at 0.5 nm intervals from 190 nm to 240 nm with 1 s intervals and 1 nm bandwidth. Data from five scans were averaged to create the CD spectra, and the secondary structures were quantified using the Provencher & Glockner Method [36].

### 4.9. Cell-Culture

WEHI-274.1 murine monocytes were cultured in DMEM supplemented with 4.5 g/L glucose, 10% *v*/*v* FBS, 1% penicillin-streptomycin, and 0.05 mM 2-mercaptoethanol. Monocytes were cultured in 5% CO_2_ and cells at passage five were used for assays.

### 4.10. In Vitro Biocompatibility

To determine the compatibility of PAMs with monocytes, 1 µM, 10 µM, and 100 µM of S-MCP-1 PAMs, S-Scrambled PAMs, C-MCP-1 PAMs, C-Scrambled PAMs, MCP-1 peptides, and scrambled MCP-1 peptides were incubated with WEHI-274.1 monocytes (4000 cells/well). Cell viability was determined at 24 h using a (3-(4,5-dimethylthiazol-2-yl)-5-(3-carboxymethoxyphenyl)-2-(4-sulfophenyl)-2H-tetrazolium) MTS Assay (BioVision, Milpitas, CA, USA). The IC_50_ values were measured by analyzing cell viability % vs. concentration, and the 100% viability represented non-treated monocytes (positive control).

### 4.11. Binding Assay

To quantify the monocyte-binding capabilities of S- or C-MCP-1 PAMs, WEHI-274.1 monocytes were seeded at 500,000 cells per well in a 6-well plate and incubated with 100 µM PAMs or PBS for 1 h at 37 °C. Cells were washed with PBS, fixed on glass microscope slides using 4% paraformaldehyde and observed under a confocal microscope (Confocal Laser Scanning Microscope 780, Zeiss, Thornwood, NY, USA) at 750 nm.

### 4.12. Chemotaxis Assay

To determine the chemoattractant properties of PAMs, 50,000 monocytes in 20 µL were suspended on a 24-well transwell plate with 8 µm pores. After 4 h, the number of cells that migrated to the bottom well containing 1 µM, 10 µM, 100 µM, 1000 µM PAMs, peptides, or PBS was quantified by lysing the cells via sonication, and measuring DNA using Quant-it Pico Green (Invitrogen, Carlsbad, CA, USA).

### 4.13. Statistical Analysis

A student’s *t*-test was conducted to compare means of pairs, and analysis of variance (ANOVA) with Tukey multiple comparison test post-hoc analysis was used to determine statistical differences among three or more means. A *p*-value less than 0.05 was considered to be statistically significant.

## 5. Conclusions

We compared spherical and cylindrical MCP-1 micelles to determine if nanoparticle shape can affect binding properties and mimic the bioactive, chemoattractant properties. Two different hydrophobic moieties were utilized in the synthesis: DSPE-PEG_2000_, which has a propensity to form spherical PAMs, and diC_16_, which forms cylindrical PAMs. Both PAMs showed enhancement in monocyte binding and no decreases in biocompatibility. Furthermore, cylindrical micelles demonstrated a significant ability to attract monocytes compared to the spherical micelles. In addition to the differences in shape, there are several distinctions between spherical and cylindrical MCP-1 PAMs that may contribute to monocyte migration and chemotaxis. Consequently, future studies will expand our investigations to understand other variables that influence bioactivity.

## Figures and Tables

**Table 1:** Dimensions, Polydispersity Index (PDI), zeta potential, and secondary structure of peptides and PAMs.

	S-MCP-1 PAM	S-Scrambled PAM	MCP-1 Peptide	Scrambled MCP-1 Peptide	C-MCP-1 PAM	C-Scrambled PAM
MW (g/mol)	5830	5830	2892	2892	3571	3571
Diameter (nm)	18.7 ± 4.0 ^a^ (14.9 ± 6.0) ^b^	18.4 ± 5.8 ^a^ (15.1 ± 7.1) ^b^			24.7 ± 6.2 ^a^ (10.7 ± 0.3) ^b^	23.7 ± 1.6 ^a^ (9.3 ± 2.0) ^b^
Length (nm)	-	-			62.2 ± 41.3	51.7 ± 15.8
PDI	0.12 ± 0.01	0.13 ± 0.03			0.11 ± 0.01	0.13 ± 0.06
Zeta Potential (mV)	1.5 ± 0.6	3.5 ± 0.5			17.6 ± 5.7	18.8 ± 3.4
Secondary Structure						
Beta Sheet (%)	50.9	46.3	36.2	43.9	55.4	55.6
Random Coil (%)	36.8	37.0	54.5	47.5	32.6	31.6
Alpha Helix (%)	12.2	16.6	9.2	8.6	8.7	12.7

^a^ Measured by dynamic light scattering (DLS) analysis; ^b^ Measured via ImageJ from TEM images.

## Supplementary Materials

The following supplementary materials are available online, Figure S1: Reaction scheme of the diC_16_ tail; Figure S2: ^1^H-NMR analysis of 1’-3’-dihexadecyl L-glutamate in CDCl_3_; Figure S3: ^1^H-NMR analysis of diC_16_ tail in CDCl_3_; Figure S4: HPLC chromatograms of peptides and conjugates; Figure S5: MALDI-TOF mass spectra of the MCP-1 peptide; Figure S6: MALDI-TOF mass spectra of the scrambled MCP-1 peptide; Figure S7: MALDI-TOF mass spectra of DSPE-PEG_2000_ MCP-1; Figure S8: MALDI-TOF mass spectra of DSPE-PEG_2000_ scrambled MCP-1; Figure S9: MALDI-TOF mass spectra of diC_16_ MCP-1; Figure S10: MALDI-TOF mass spectra of diC_16_ scrambled MCP-1; Figure S11: MALDI-TOF mass spectra of DSPE-PEG_2000_ Cy7; Figure S12: MALDI-TOF mass spectra of diC_16_ Cy7; Figure S13: Dynamic light scattering (DLS) size intensity of PAMs; Figure S14: Circular dichroism spectroscopy of PAMs and peptides; Figure S15: Particle stability of S- and C-MCP-1 PAMs.
