# Supplementary material for: Shape Effects of Peptide Amphiphile Micelles for Targeting Monocytes
Source: Molecules. 2018 Oct 27;23(11):2786. doi: 10.3390/molecules23112786 (PMC6278295; doi:10.3390/molecules23112786)
Supplement: Supplementary file 1 [file molecules-23-02786-s001.pdf]

molecules

MDPI

Article

# Shape Effects of Peptide Amphiphile Micelles for Targeting Monocytes

**Johan Joo [1,†], Christopher Poon [1,†], Sang Pil Yoo [2,3] and Eun Ji Chung [1,4,5,6,7,*]**

1 Department of Biomedical Engineering, University of Southern California, Los Angeles, CA 90089, USA; johanjoo@usc.edu (J.J.); poonc@usc.edu (C.P.)
2 Institute for Molecular Engineering, University of Chicago, 5747 South Ellis Avenue, Chicago, Il 60637, USA; sangpilyoo@mednet.ucla.edu
3 Current Affiliation: Medical Scientist Training Program, David Geffen School of Medicine, University of California, Los Angeles, Los Angeles, CA 90096, USA
4 Mork Family Department of Chemical Engineering and Materials Science, University of Southern California, Los Angeles, CA 90089, USA
5 Division of Nephrology and Hypertension, Department of Medicine, Keck School of Medicine, University of Southern California, Los Angeles, CA 90033, USA
6 Norris Comprehensive Cancer Center, University of Southern California, Los Angeles, CA 90033, USA
7 Department of Stem Cell Biology and Regenerative Medicine, University of Southern California, Los Angeles, CA 90033, USA
* Correspondence: eunchung@usc.edu; Tel.: +1-213-740-2925
† These authors contributed equally to this work.

## Supplemental Figures

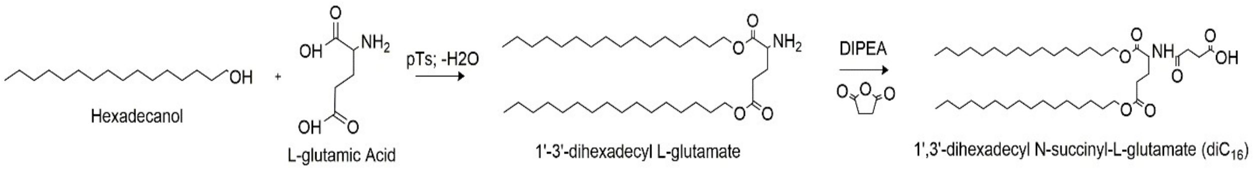


**Figure S1.** Synthesis of 1'-3'-dihexadecyl L-glutamate through an azeotropic distillation. The resulting product was deprotonated and a spacer molecule (succinic anhydride) was inserted to form the diC$_{16}$ tail.

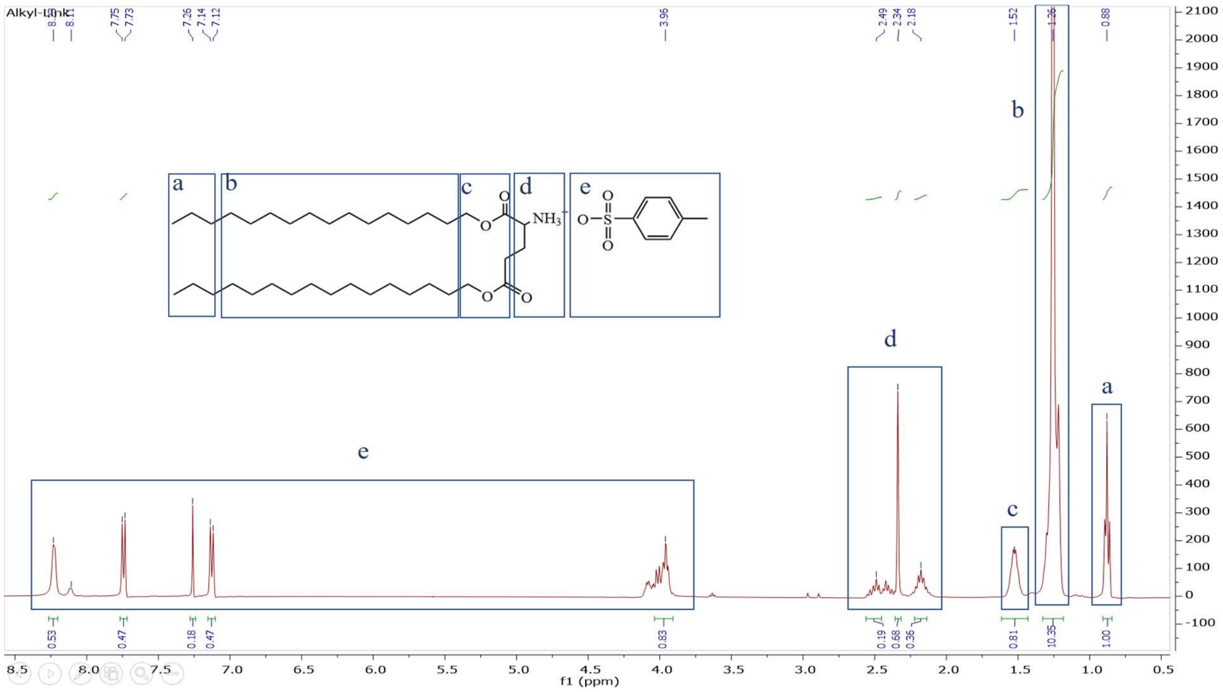


**Figure S2.** $^1$H-NMR analysis of 1'-3'-dihexadecyl L-glutamate in CDCl$_3$. The peaks within boxes correspond to a specific part of the compound structure.

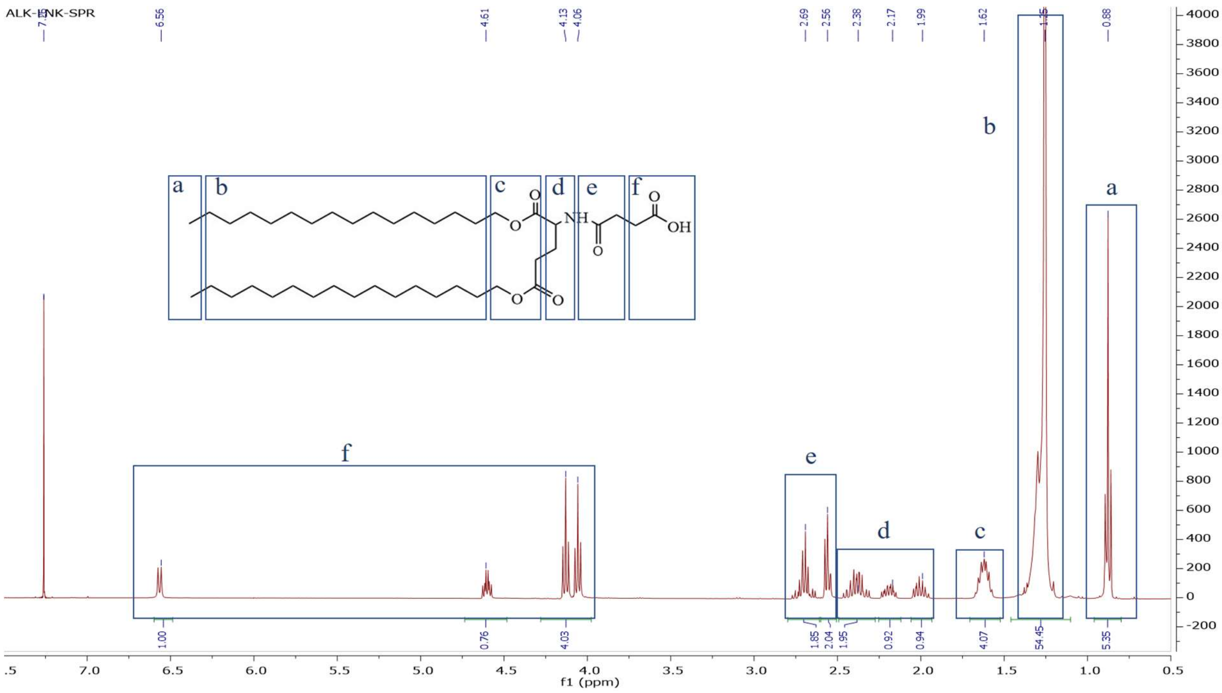


**Figure S3.** $^{1}$H-NMR analysis of diC$_{16}$ tail (1',3'-dihexadecyl N-succinyl-L-glutamate) in $CDCl_3$. The peaks within boxes correspond to a specific part of the compound structure.

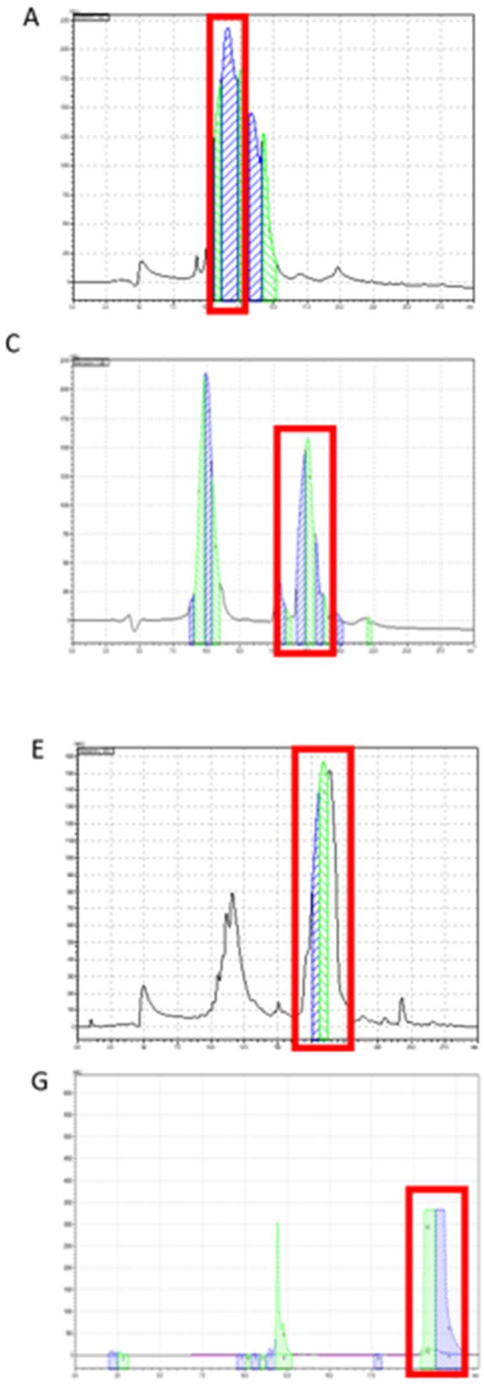


**Figure S4.** HPLC chromatogram of A) MCP-1 peptide, B) Scrambled peptide, C) DSPE-$PEG_{2000}$-MCP-1, D) DSPE-$PEG_{2000}$-Scrambled, E) diC16 MCP-1, F) diC16 Scrambled, G) DSPE-$PEG_{2000}$-Cy7, and H) diC16-Cy7. The highlighted area (red box) were analyzed via MALDI (Figure S5-S12).

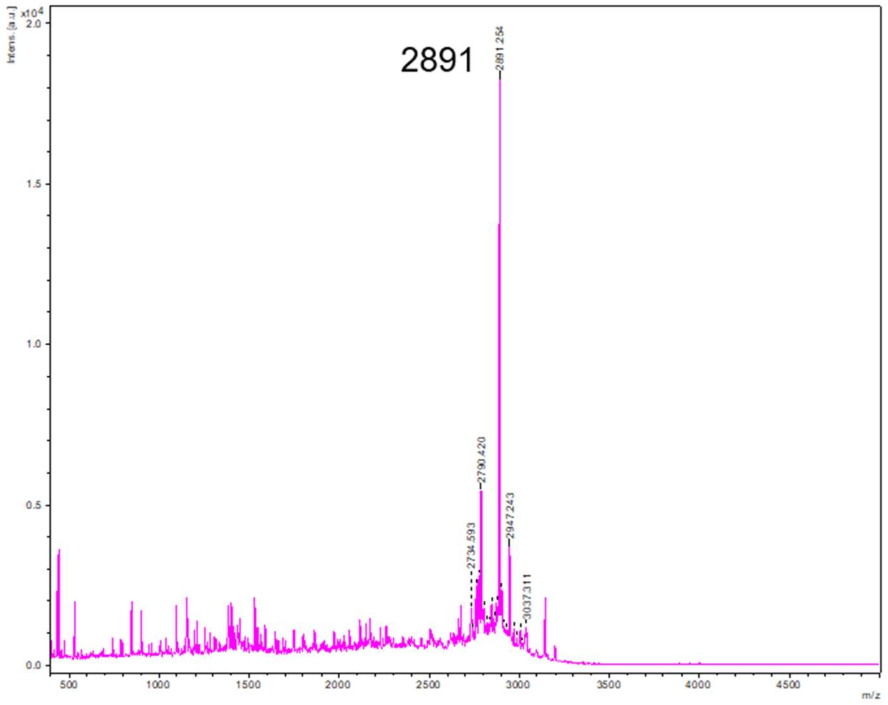


**Figure S5.** MALDI-TOF mass spectra of the MCP-1 peptide at a range of 400 – 5000 Da. Highest peak corresponds to $[M + H]^+$ = 2891 (expected 2892).

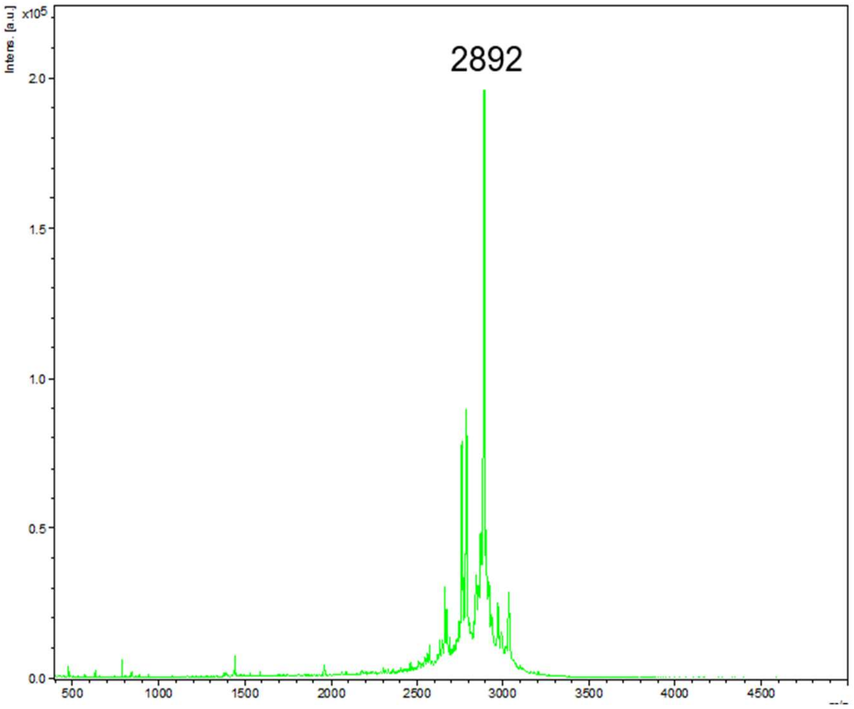


**Figure S6.** MALDI-TOF mass spectra of the scrambled MCP-1 peptide at a range of 450 – 5000 Da. Highest peak corresponds to $[M + H]^+$ = 2892 (expected 2892).

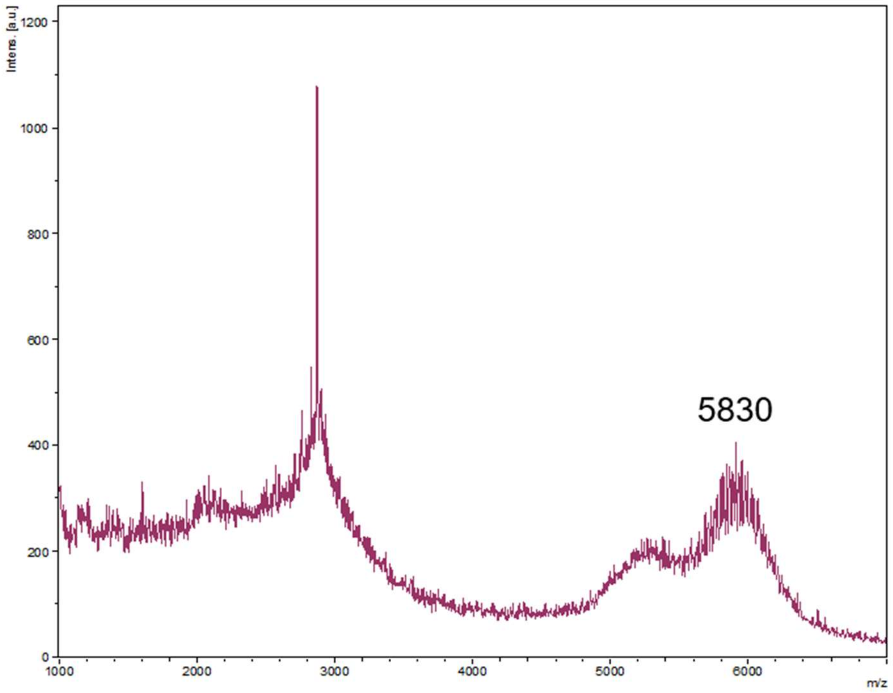


**Figure S7.** MALDI-TOF mass spectra of DSPE-PEG$_{2000}$ MCP-1 at a range of 1000 – 7000 Da. Rightmost peak corresponds to $[M + H]^+$ = 5830 (expected 5830).

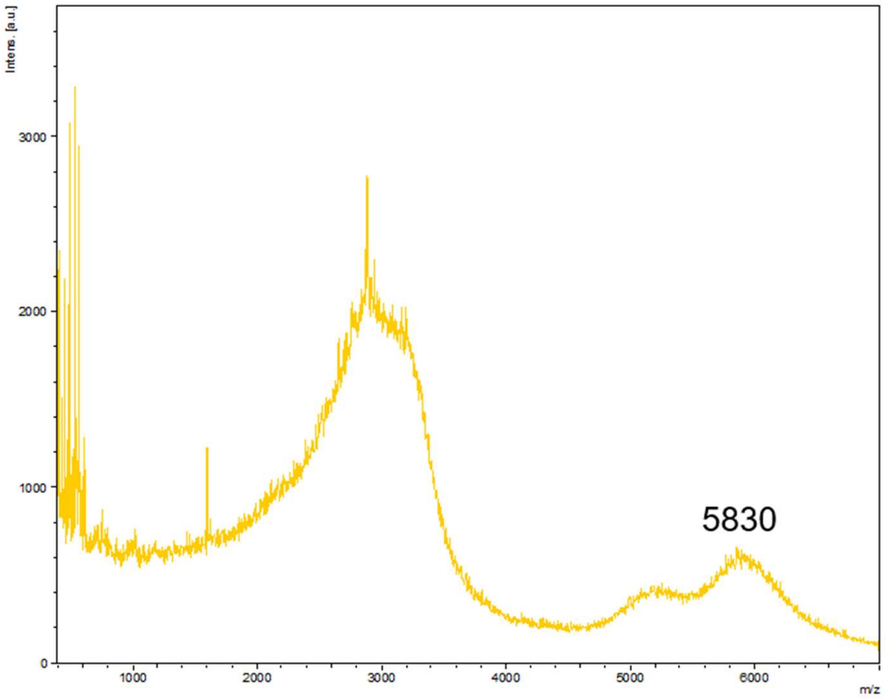


**Figure S8.** MALDI-TOF mass spectra of DSPE-PEG$_{2000}$ scrambled MCP-1 at a range of 400 – 7000 Da. Rightmost peak corresponds to $[M + H]^+$ = 5830 (expected 5830).

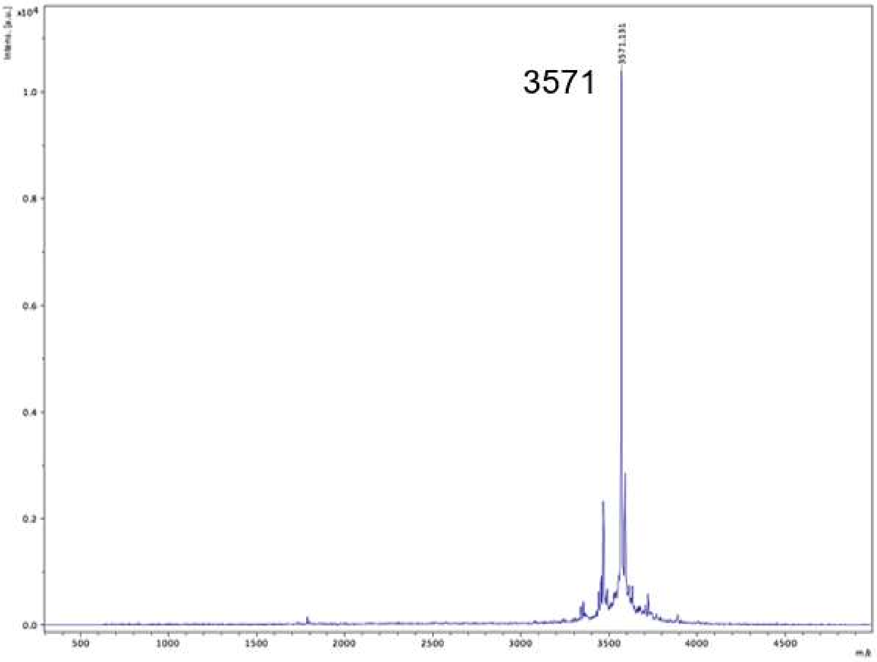


**Figure S9.** MALDI-TOF mass spectra of diC$_{16}$ MCP-1 at a range of 300 – 5000 Da. Highest peak corresponds to $[M + H]^+$ = 3571 (expected 3571).

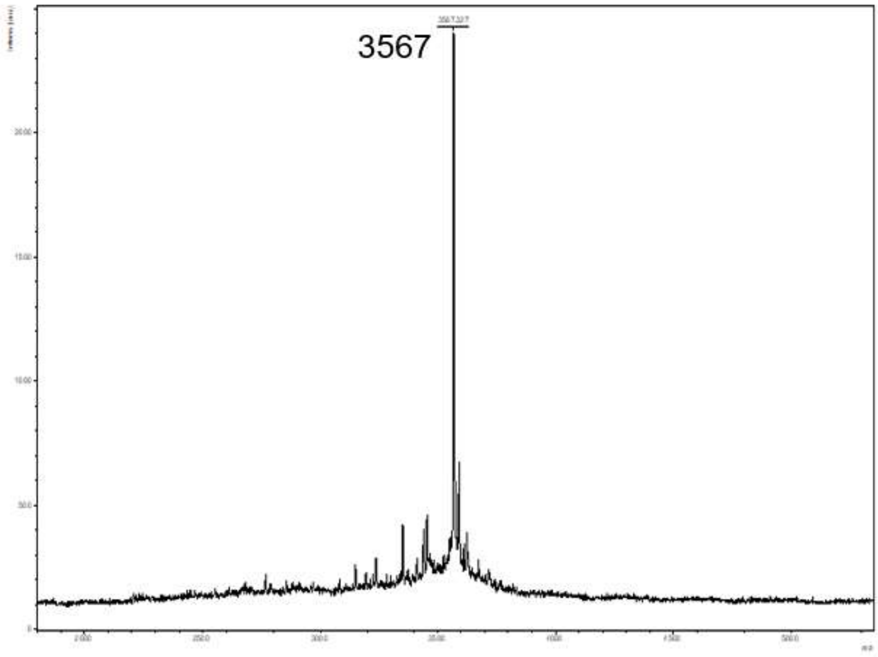


**Figure S10.** MALDI-TOF mass spectra of diC$_{16}$ scrambled MCP-1 at a range of 1800 – 5400 Da. Highest peak corresponds to $[M + H]^+$ = 3567 (expected 3571).

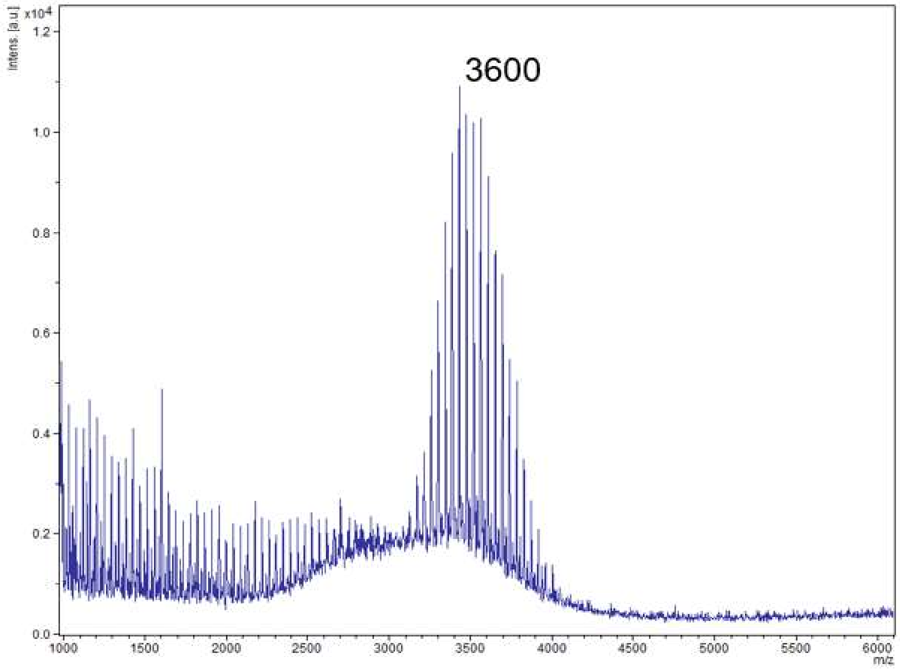


**Figure S111.** MALDI-TOF mass spectra of DSPE-PEG$_{2000}$ Cy7 at a range of 1000 – 6100 Da. Center peak corresponds to $[M + H]^+$ = 3600 (expected 3680).

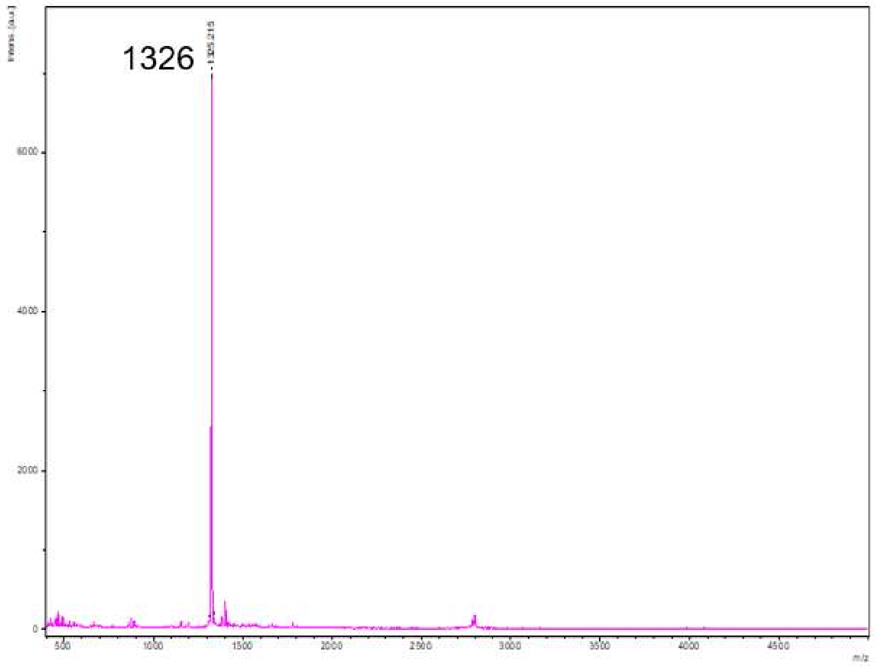


**Figure S12.** MALDI-TOF mass spectra of diC$_{16}$ Cy7 at a range of 400 – 5000 Da. Highest peak corresponds to $[M + H]^+$ = 1326 (expected 1326).

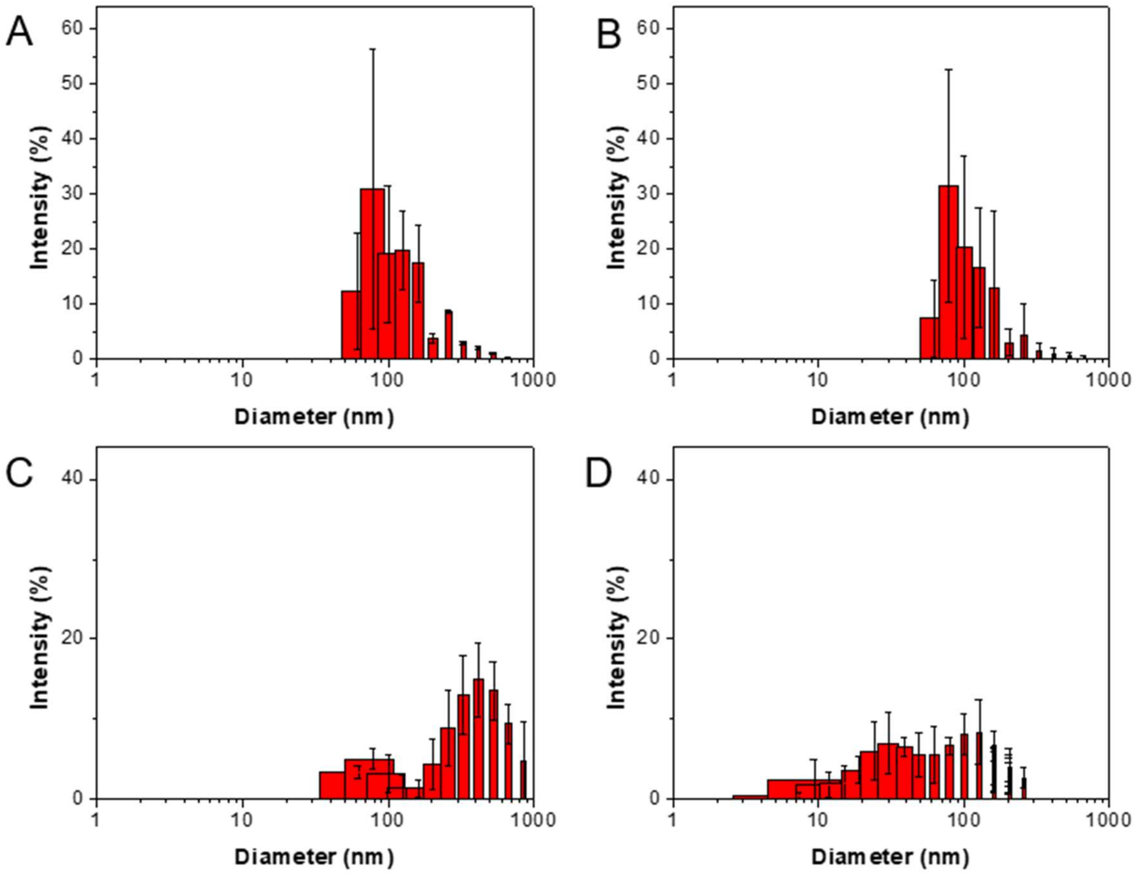


**Figure S13.** Dynamic light scattering (DLS) size intensity averages of A) S-MCP-1 PAMs, B) S-Scrambled PAMs, C) C-MCP-1 PAMs, and D) C-Scrambled PAMs.

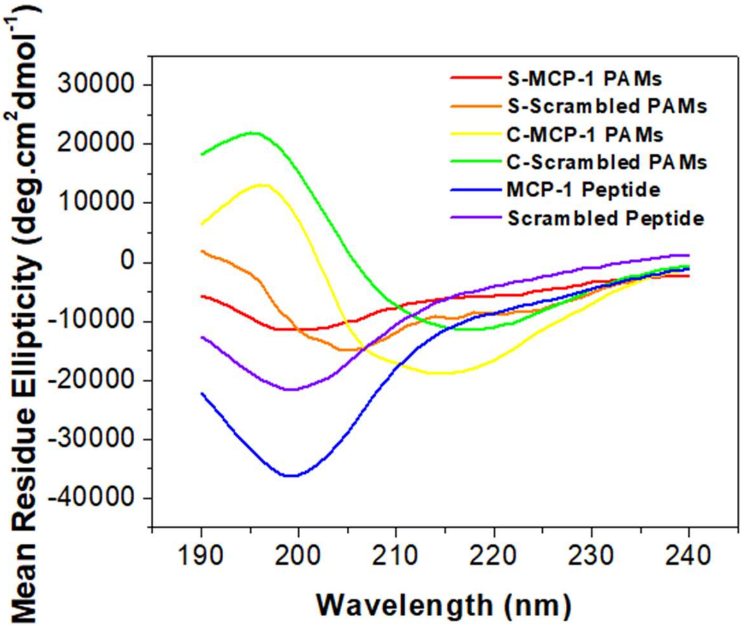


**Figure S14**. Circular dichroism spectroscopy of spherical MCP-1 PAMs (DSPE-PEG$_{2000}$ MCP-1 PAMs), scrambled spherical PAMs, cylindrical MCP-1 PAMs (diC$_{16}$ MCP-1 PAMs), scrambled cylindrical MCP-1 PAMs, MCP-1 peptides, and scrambled peptides.

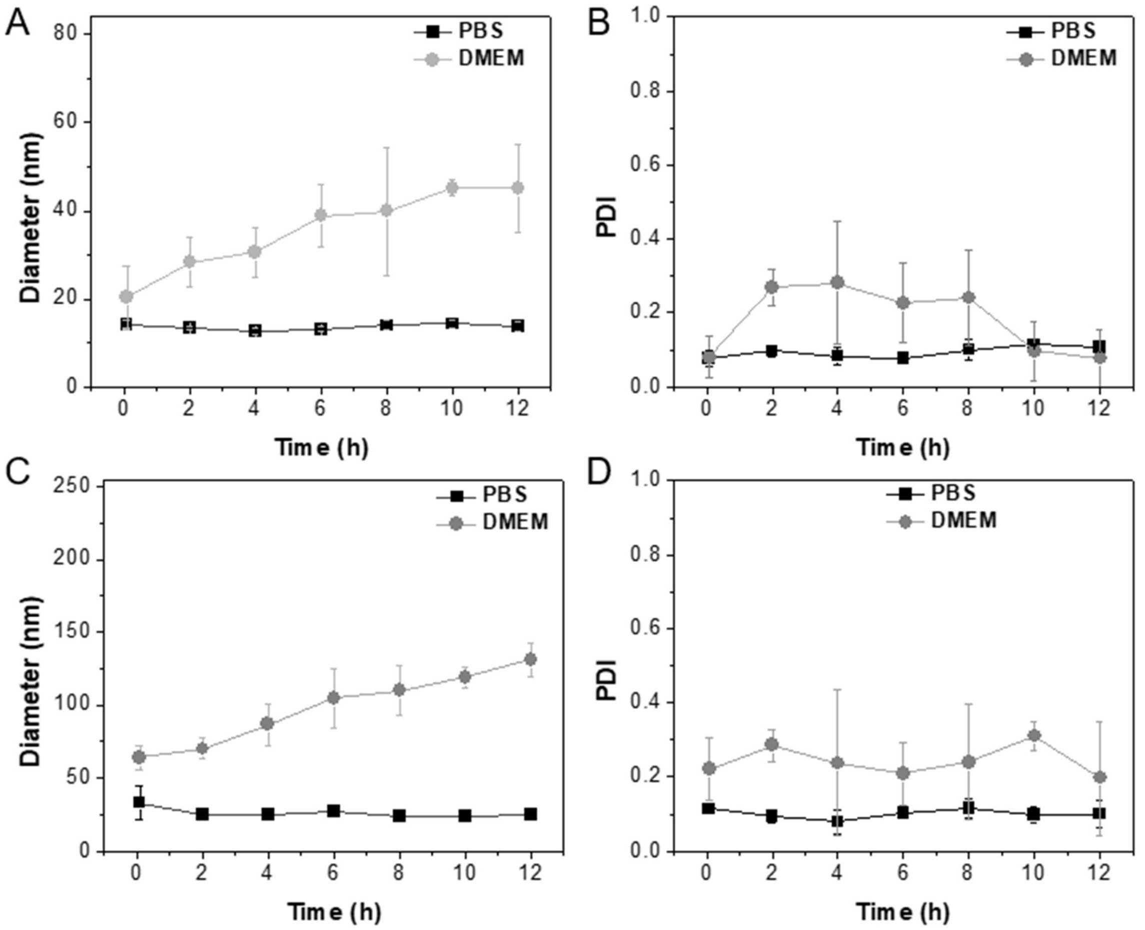


**Figure S15.** Particle stability of A) S-MCP-1 PAMs and C) C-MCP-1 PAMs measured by DLS, along with the polydispersity index (PDI) of B) S-MCP-1 PAMs and D) C-MCP-1 PAMs.
